# Evaluation of the C_60_ biodistribution in mice in a micellar *ExtraOx* form and in an oil solution

**DOI:** 10.1038/s41598-021-87014-3

**Published:** 2021-04-16

**Authors:** Konstantin N. Semenov, Daria A. Ivanova, Sergei V. Ageev, Andrey V. Petrov, Nikita E. Podolsky, Ekaterina M. Volochaeva, Ekaterina M. Fedorova, Anatolii A. Meshcheriakov, Egor E. Zakharov, Igor V. Murin, Vladimir V. Sharoyko

**Affiliations:** 1 Pavlov First Saint Petersburg State Medical University, L’va Tolstogo ulitsa 6–8, Saint Petersburg, Russia 197022; 2 Institute of Chemistry, Saint Petersburg State University, Universitetskii prospect 26, Saint Petersburg, Russia 198504; 3 AQUANOVA RUS” JSC, Prospekt Nauki 12, Dubna, Moscow Oblast Russia 141983; 4 A. M. Granov Russian Research Centre for Radiology and Surgical Technologies, 70 Leningradskaya ulitsa, Saint Petersburg, Russia 197758

**Keywords:** Biomedical materials, Carbon nanotubes and fullerenes, Nanostructures

## Abstract

The article is devoted to the study of the pharmacokinetics of fullerene C_60_ in oil and micellar forms, analysis of its content in blood, liver, lungs, kidneys, heart, brain, adrenal glands, thymus, testicles, and spleen. The highest accumulation of C_60_ was found in the liver and adrenal glands. As a result of the studies carried out, it was shown that the bioavailability of C_60_ in the micellar form is higher than that in an oil solution.

## Introduction

Attention to fullerenes as a basis for the design of biologically active substances is determined by their following properties: size, lipophilicity, structure (presence of internal volume), chemical and photophysical properties. The molecular sizes of C_60_ and C_70_ fullerenes are 0.714 and 0.780 nm, respectively. Hence, fullerene molecules can complementarily interact with biological targets. It is this complementary interaction of the fullerene core with the cavity in the virus-specific protease that underlies the mechanism of action of HIV protease inhibitors^1,2^.

Non-modified C_60_ fullerene molecule is highly lipophilic, its octanol–water partition coefficient (log*P*_ow_) is 6.67^3^. The value of log*P* for such a lipophilic substance as xylene is 3.20, which once again underlines the high lipophilicity of fullerene. If the value of log*P* > 0–3, then such substances quickly pass through the membrane and are quickly distributed throughout the body. A further increase in this value reduces adsorption, as a result, the substance more difficult to penetrate into the cell membrane. Moreover, a substance with high lipophilicity, getting into the membrane, remains there, and is not further distributed throughout the body. The high affinity of fullerenes for lipophilic systems determines the membranotropic mechanism of antiviral action, and also underlies the creation of sorbents for affinity chromatography of proteins^4–6^. For example, in ref.^7^, systems for the transfer of proteins enriched with L-arginine, which are able to penetrate through bilayer membranes, were developed using the fullerene derivative C_60_(COOH)_2_. The authors of refs.^8,9^ showed that conjugates of C_60_ with N-fluorescein-5-isothiocyanate pyrrolidine and doxorubicin interacted with the lipid matrix of the cell membrane and, due to the membranotropic action of C_60_, transmembrane transport of the conjugates occurs.

Of particular interest is the presence of an internal volume in fullerene molecules, their ability to form endohedral metallofullerenes (EMF)^10–14^. Functionalised EMF can be used in the following means of therapy: photodynamic therapy (PDT)^15^, hyperthermia, radiotherapy, and chemotherapy^16,17^.

The photophysical and chemical properties of fullerene are determined by the presence of a system of conjugated double bonds in the molecule, i.e. are a fundamental property of the fullerene core itself. For example, fullerene molecules under the action of visible or UV radiation promote the conversion of triplet oxygen into singlet oxygen^18–20^, thereby acting as a singlet oxygen “generator”. This is due to the fact that the C_60_ fullerene molecule in the ground state (^1^C_60_), upon irradiation with UV and visible light, easily transforms into an excited singlet state (^1^C_60_*), which, as a result of a transition with a high quantum yield (about 100%), turns into an excited triplet state (^3^C_60_*). In turn, the excited triplet state (^3^C_60_*) transfers energy to the molecule of triplet oxygen ^3^O_2_, converting it into singlet oxygen ^1^O_2_^21,22^. Separately, mention should be made of a series of works in which the effectiveness of composites based on C_60_ immobilised at nano-silica particles, as well as C_60_–cisplatin nanocomplexes for photodynamic therapy of oncological diseases was shown^23–25^.

Since in biological systems reactions with the participation of free radicals are mainly oxidation reactions, then one of the biological properties of fullerene C_60_ can be antioxidant activity^26–30^. In ref.^31^, results were obtained indicating a relationship between the antioxidant effect of fullerene and lifespan of rats. Oral administration of a solution of fullerene in olive oil increased the lifespan of *Wistar* rats, and the authors attribute this to the antioxidant properties of fullerene. Due to these properties, fullerenes have found their application in cosmetology and dermatology. On the market for fullerene cosmetics, Japanese manufacturers are leading: the most successful company is Vitamin C_60_ Research Corporation (a subsidiary of Mitsubishi Corporation), which produces the most common fullerene-containing products, RadicalSponge or Lipofullerene. According to Vitamin C_60_ Research Corporation, more than 1,500 clinics in Japan use products in which fullerene serves as an active ingredient, and it is believed that there are more than 1000 such products. Compositions based on them act as nanotraps for radicals without causing toxic effects^32–34^.

Due to the low solubility of fullerenes in water (< 10^−11^ g·l^−1^)^35,36^, there is a problem of their study and application in medicine. An alternative way of injecting fullerenes into the body is to use solutions in such solvents as natural oils and animal fats. These systems have many advantages including:

- fullerenes are decently soluble in these natural solvents from tenths to several grams of fullerenes per litre of a solution^37–41^;
- fullerenes form absolutely transparent true solutions stable in time with natural vegetable oils and animal fats^42^;
- such solutions are completely harmless and compatible with organisms of animals and humans, if they are prepared directly during the extraction of a fullerene mixture from fullerene soot by oils themselves;
- fullerene solutions in oils and fats have pronounced bactericidal and antioxidant properties; they can also scavenge free radicals and radical ions from condensed phases, as well as photons in the UV region of the spectrum^43,44^.

Speaking about the specific toxicological effects of fullerene on a living organism, it is worth paying attention to ref.^45^, where the effect of an aqueous suspension of C_60_ was studied when administered intraperitoneally into *Sprague–Dawley* rats. No acute toxicity was found; on the contrary, it turned out that fullerene exhibits hepatoprotective properties in the modelled CCl_4_-induced hepatitis. Also, no toxicological effects were found in the study of the C_60_ complex with polyvinylpyrollidone^46,47^.

Ref.^48^ was devoted to the toxic effects of fullerene. Fullerene C_60_ was administered perorally to mice (female and male, age: 4 weeks) in corn oil, once a day at doses of 1, 10, 100 or 1000 mg·kg^−1^ per day for 29 days, followed by a 14-day recovery period. The study did not reveal any toxic effects of C_60_ fullerene, but there was a slight increase in liver and spleen mass after a 14-day recovery period, which may be associated with the effect of oral intake of C_60_ fullerene. Experiments on acute toxicity showed that administration of C_60_ fullerene aqueous colloid solution in a concentration range of 75–150 mg·kg^−1^ to mice did not cause toxic effects. In an in vitro experiment, C_60_ fullerene in the concentration range of 3.6–144 mg·ml^−1^ was found to have low toxicity towards human embryonic kidney line (HEK293), and the *IC*_50_ value was 383.4 μg·ml^−1^^49^. Ref.^50^ revealed that fullerene C_60_ can stimulate the growth of cervical cancer cell line HeLa and human mesenchymal stem cells at low concentrations (6–12 μg·ml^−1^) and reduce cell viability at high concentrations (24 μg·ml^−1^).

This article is devoted to the study of the pharmacokinetics (PK) of C_60_. The relevance of the study is associated with the fact that it allows obtaining information on the biodistribution and bioavailability. The study of bioavailability reveals the organs and tissues in which fullerene penetrates most actively and accumulates, which can contribute to a more detailed understanding of the mechanisms of action of fullerene.

## Experimental part

### Materials

**Figure 1:**
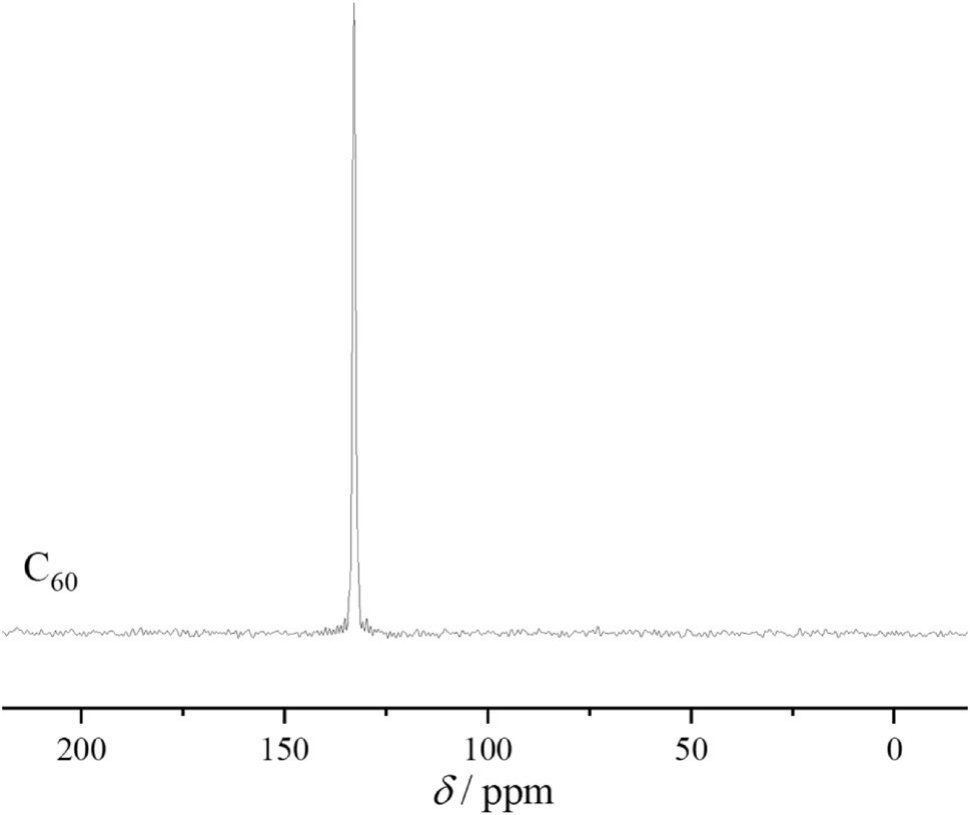
^13^C NMR spectrum of C_60_ obtained with CP/MAS technique.

A sample of olive oil (All-Russian Scientific Research Institute of Fats) of the following composition (wt.%): palmitic acid (7.00–20.00), palmitoleic acid (0.3–3.5), stearic acid (1.5–4.3), oleic acid (56.0–86.0), linoleic acid (3.3–20.0), linolenic acid (0.4–1.5), arachidonic acid (0.2–1.6), gondoic acid (0.2–0.5), toluene 99.9% (Sigma-Aldrich) without additional purification, fullerene C_60_ 99.9%, produced by MST-NANO (St. Petersburg). For additional identification of fullerene, a number of physicochemical methods were used: NMR spectroscopy (NMR spectrometer Bruker Avance III 400 MHz). During the NMR study, the sample was placed in a rotor with an outer diameter of 4 mm, made of zirconium oxide and a rotation frequency of 12.5 kHz at a magic angle to the direction of a constant magnetic field. To record the spectra on ^13^C nuclei, a cross-polarisation sequence of exciting pulses (CP/MAS technique) was used; the contact time was 2,000 ms. The resulting spectrum (Fig. 1) contains one peak, which is related to the carbon atoms of the fullerene core.

**Figure 2:**
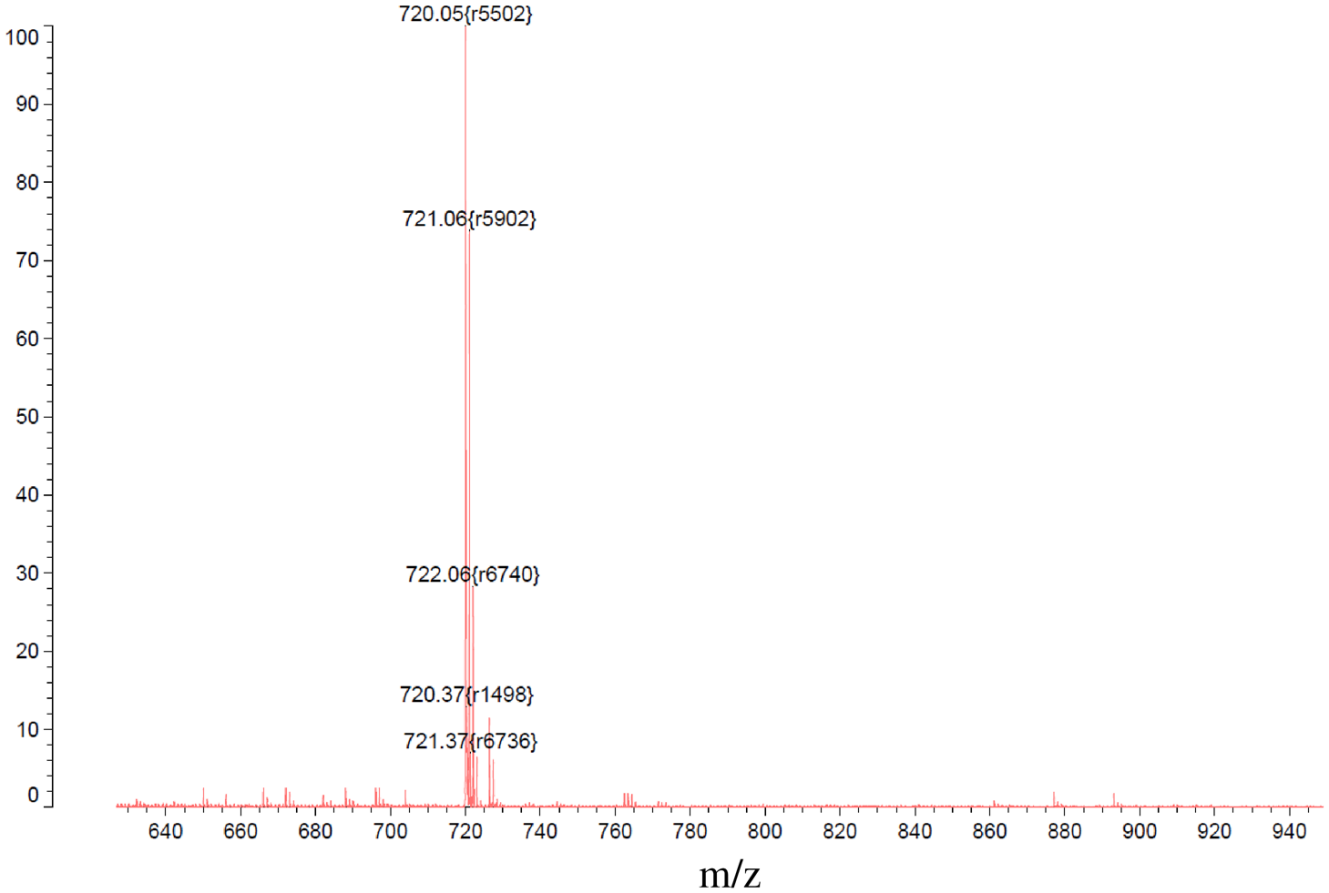
MALDI mass spectrum of C_60_ fullerene.

Figure 2 shows the mass spectrum of a C_60_ fullerene sample obtained by negative ionisation using a Muldi mass spectrometer (Shimadzu Axima—Resonance). The presented spectrum shows a set of peaks corresponding to the C_60_ isotopic distribution, which indicates the absence of impurities in the sample.

**Figure 3:**
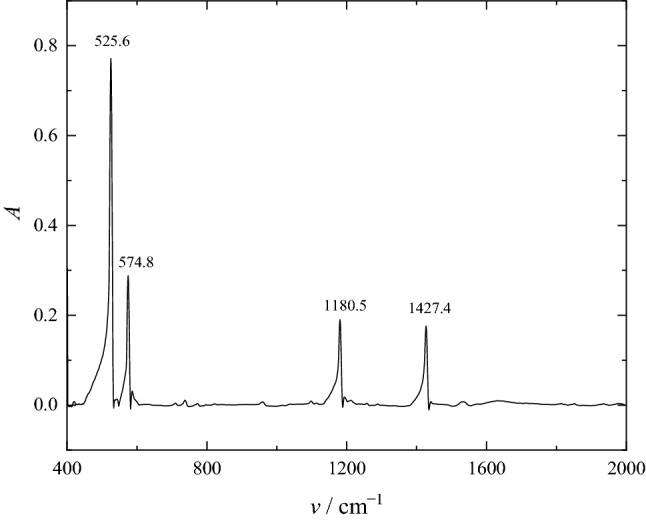
IR spectrum of C_60_ fullerene.

The transmission spectrum was recorded on a Shimadzu apparatus, after mixing the investigated powdered fullerene C_60_ with microcrystalline potassium bromide (KBr) and subsequent pressing of the microtablet from this mixture. The spectrum (Fig. 3) shows bands at 1427.4, 1180.5, 574.8, 525.6 cm^−1^, caused by vibrations of free bonds of fullerene C_60_. The data obtained are in good agreement with the literature ones^51,52^.

**Figure 4:**
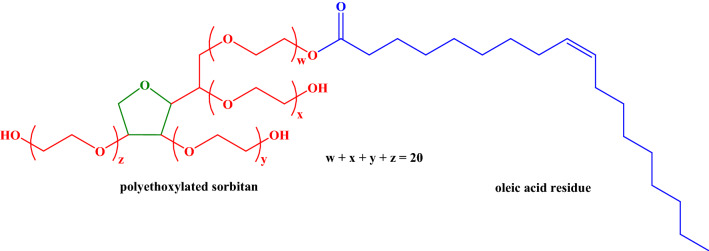
A chemical structure of TWEEN-80.

When studying the pharmacokinetics, the oil and micellar forms of fullerene C_60_ were used. A micellar solution of fullerene C_60_ contained an oil solution of C_60_ fullerene, as well as a surfactant TWEEN-80 purchased from Sigma-Aldrich (molecular weight 1310 g·mol^−1^) approved for the use in cosmetic, pharmaceutical, and food industry and applied to form micelles. Moreover, it was determined that this surfactant does not possess cancerogenic and mutagenic activity. TWEEN-80 is a non-ionic surfactant derivative of polyethoxylated sorbitan and oleic acid (Fig. 4). Its hydrophilic-lipophilic balance value is 16.7 which determines the hydrophilicity of that kind of a surfactant, and consequently an ability to stabilise an oil-in-water emulsion. In addition, micellar form is more bioavailable in comparison to oil one. The micellar form of fullerene in oil was obtained using the original ExtraOx technology developed by “AQUANOVA RUS” JSC^53^. This technology makes it possible to obtain micelles up to 30 nm in size, in which various biologically active substances are encapsulated in a shell consisting of surfactants. The structure of such a micelle mimics the micelle that is formed in the human body when lipids are absorbed during digestion. Due to the creation of micellar forms of various biologically active substances, the bioavailability of the active ingredient is significantly increased, as well as the stability during storage is improved.

The content of fullerene C_60_ in the oil form is 0.1 wt.%, in the micellar form is 0.01 wt.%.

**Figure 5:**
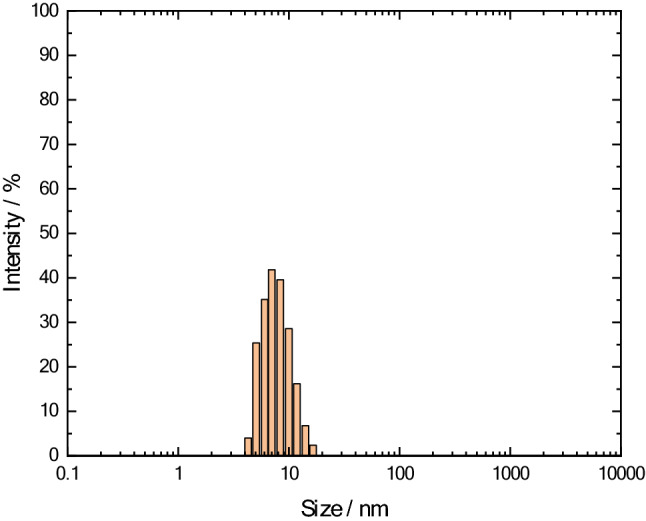
Size distribution of a C_60_ micellar form.

The distribution of C_60_ micellar particles was studied by dynamic light scattering on a Malvern Zetasizer 3000 device (Malvern Instruments, Malvern, Worcestershire, United Kingdom). The obtained results are presented in Fig. 5. It can be seen that the average micelle size is 10 ± 5 nm.

### Equipment and research methods

#### Study of the solubility of C_60_ in olive oil

To determine the saturation time of fullerene solutions, the kinetics of fullerene dissolution was studied. The determination of the polythermal solubility of C_60_ in olive oil was carried out using the isothermal saturation method. According to this method, olive oil was added to the flask with a notorious excess of C_60_. Then the flask was thermostated (thermostating accuracy 0.1 °C) by shaking at a frequency of up to 3.5 cycles per second for a time sufficient for saturation. The time required to establish a stationary concentration value was 20 h.

**Figure 6:**
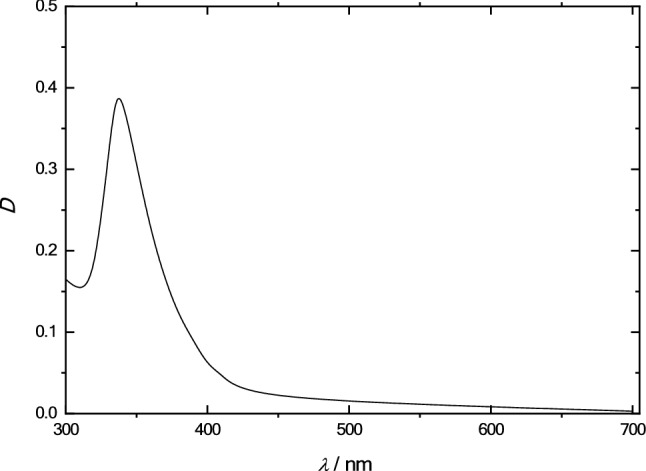
Absorption spectrum of fullerene C_60_ in olive oil.

Concentrations were determined by means of SOLAR CM 2203 spectrofluorometer (Solar CJSC, Minsk, Belarus). As is known, fullerene C_60_ has a characteristic transmission spectrum with well-pronounced absorption maxima in the near UV and visible regions (300–600 nm)^54–58^. The obtained spectrum of fullerene C_60_ (Fig. 6) shows an almost complete absence of solvatochromic effects, which makes it possible to use reliably the following formula^42^:

1 $$C\left( {C_{60} } \right) \, = \, 13.10 \cdot \left( {D_{335} - \, 1.8051 \cdot D_{472} } \right),$$

where *D*_335_ and *D*_472_ are optical densities of the solution reduced to an absorbing layer of 1 cm, *C*(C_60_) is a fullerene concentration (mg·l^−1^).

**Figure 7:**
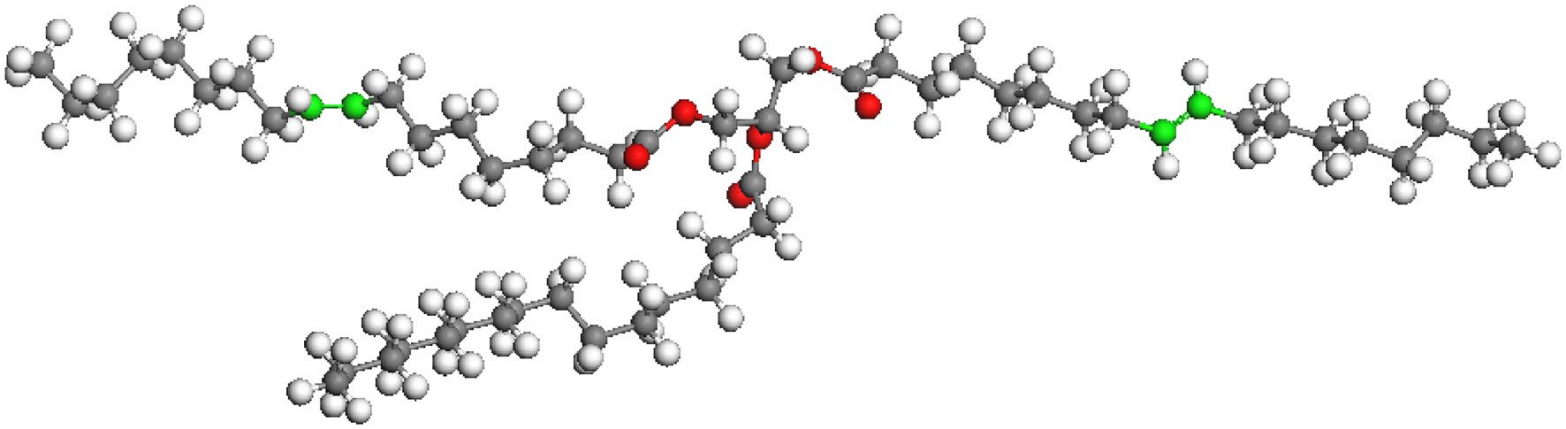
Triglyceride molecule consisting of two oleic acid residues and one palmitic acid residue.

**Figure 8:**
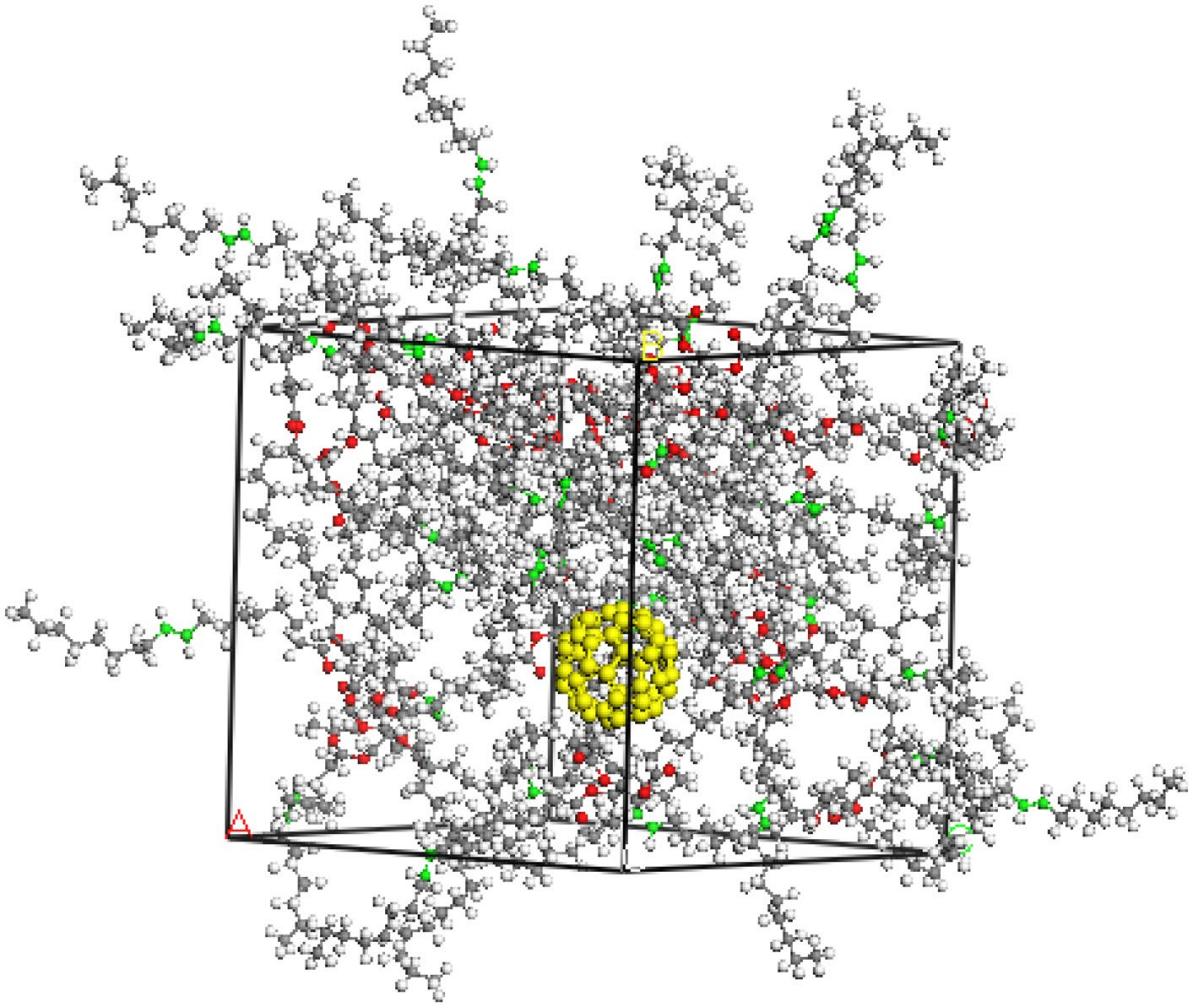
Cell with fullerene molecule and 20 triglyceride molecules.

Modelling of the structural and dynamic properties of a fullerene solution in olive oil was carried out by the molecular dynamics (MD) method. To analyse the interaction of a fullerene molecule and olive oil components in solution, a model system was chosen containing one fullerene molecule and 20 triglyceride molecules (Fig. 7), composed of two residues of oleic acid and one residue of palmitic acid. At the first stage, the electronic structure and equilibrium geometry of the triglyceride molecule were calculated by the DFT method in the DMol^3^ program from the Materials Studio package (PBE functional, basis DNP 4.4); then the charges on the atoms were determined according to the Mulliken scheme. For calculations by the method of MD, a cell was formed (Fig. 8), consisting of a fullerene molecule and triglyceride molecules, with the calculated charges. The calculations were carried out in the Forcite program (UFF force field) from the Materials Studio package. At the first stage, an integration step of 1 fs, time 1000 ps, NPT ensemble was used. A system was obtained with good agreement with the experimental density value of olive oil. At the second stage, an integration step of 0.1 fs, a time of 100 ps, an NVT ensemble was used. Thus, at each stage, 1 million steps were simulated.

The data on the solvent content in crystal solvates were obtained as follows: the solid phase freshly precipitated from the corresponding solution was washed twice with ethyl alcohol, then dried at 20 °C for 30 min, after which the resulting solid phase was weighed. After that, the solid phase was repeatedly washed in a Soxhlet apparatus with ethyl alcohol (at 78 °C, 1 atm.) and dried in a vacuum (0.1 mm Hg) at a temperature of 200 °C for 1 h, and then weighed again. The change in the mass of the solid phase was used to determine the solvent content in the initial crystal solvate.

#### Study of pharmacokinetics

The study was approved by the Local Ethics Committee of the Pavlov First Saint Petersburg State Medical University, and the followed procedures were in accordance with University guidelines and ethical standards. All experiments were complied with ARRIVE guidelines.

In the experiment, mice from the Rappolovo nursery were used: males weighing 30 ± 3 g, a total of 54 pcs. 12 h before the experiment, the animals were starved, without restricting access to water. The method of administration of the test solutions was the following: once 15 mg·kg^−1^ for the oil form of C_60_ and 1.5 mg·kg^−1^ for the micellar one. Both forms were administered intragastrically via gavage. The dose of C_60_ in oil was chosen according to ref.^59^. Preliminary experiments on the accumulation of C_60_ in oil and in the micellar form in HEK293 cells showed that the micellar form has almost an order of magnitude more accumulation in cells.

Mice were sacrificed 24 h after administration of the final dose of fullerene. The tissues of the liver, lungs, kidneys, heart, brain, adrenal glands, thymus, testicles, and spleen were collected, weighed, and stored at − 80 °C. Blood samples were collected in tubes containing 5 IU·ml^−1^ heparin sodium salt.

**Figure 9:**
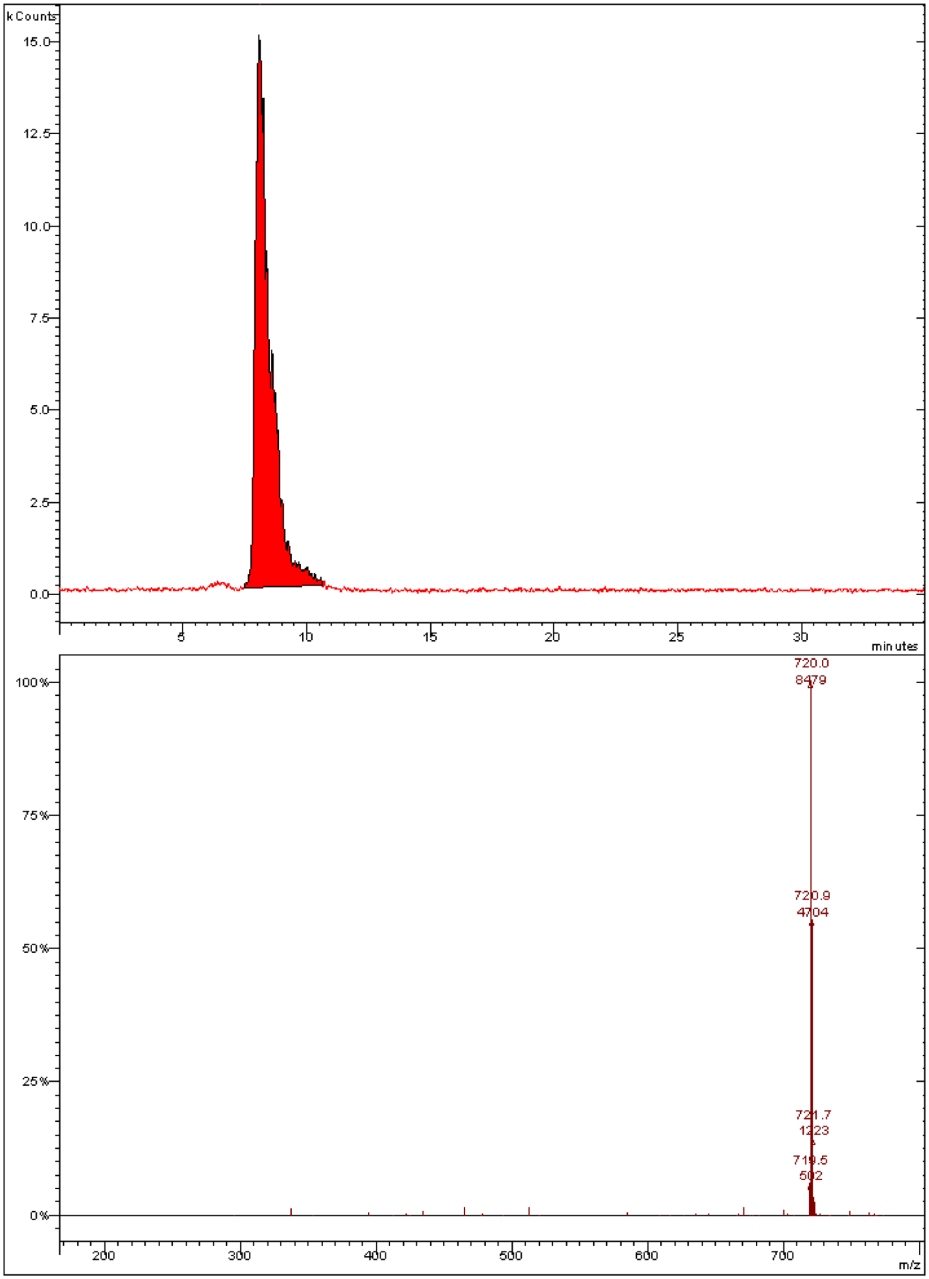
HPLC–MS chromatogram of C_60_.

A portion of the biological material was frozen and homogenised. Fullerene was extracted with 1 ml of toluene in an ultrasonic bath for 30 min. Proteins precipitated in the extracts obtained, thereafter, the extracts were centrifuged at 12,000 g for 15 min at 4 °C. The centrifugate was diluted with the mobile phase in a 1:2 ratio, re-centrifuged, and the resulting solution was analysed by electrospray ionisation gas chromatography-mass spectrometry (AgilentVarian 500-MSLC), chromatographic column Microsorb-MV 100–5 (250 mm × 4.6 mm, 5 μm, 100 A), the voltage across the capillary was − 4787 V. Figure 9 shows an example of the obtained chromatogram.

## Results and discussion

### Solubility study

**Figure 10:**
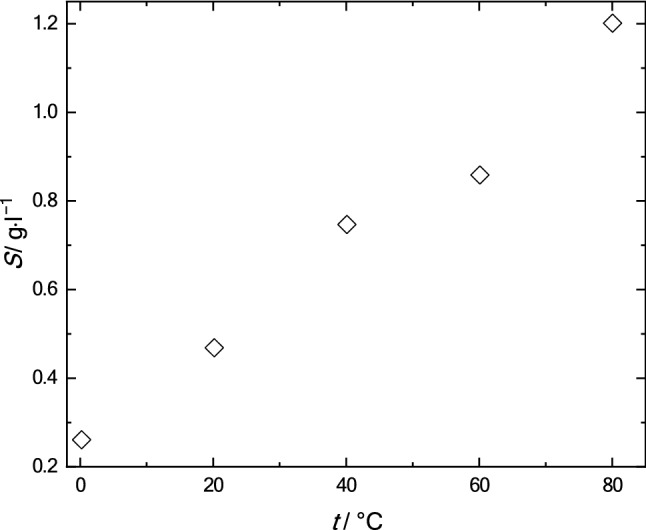
Solubility polytherm of fullerene C_60_ in olive oil in the temperature range 0–80 °C.

Analysis of the temperature dependence of solubility showed that fullerene C_60_ is quite well compatible with olive oil: solubility is from tenths to 1.2 g of fullerenes per litre of a solution in the temperature range from 0 to 80 °C (Fig. 10).

Analysis of the composition of the crystal solvate formed at relatively low temperatures (*t* ≤ 40 °C) showed that the weight loss of the crystal solvate sample after repeated washing with ethanol followed by drying in vacuum is Δ*m* ≈ 18 ± 5 rel. wt.%. The average molecular weight of the mixed triglycerides that make up the basis of olive oil is $\overline{M}$ ≈ 885 ± 15 u. In the calculation, it was assumed that one averaged triglyceride molecule in olive oil contained 2.10 oleic acid residues CH_3_(CH_2_)_7_CH=CH(CH_2_)_7_COOH, 0.45 acid residue of palmitic acid CH_3_(CH_2_)_14_COOH and 0.45 acid residue of linoleic acid CH_3_(CH_2_)_3_(CH_2_CH = CH)_2_(CH_2_)_7_COOH. Then the calculation makes it possible to obtain the values of the average composition of the crystal solvate C_60_(0.17 ± 0.05)TG (TG—a triglyceride of olive oil). Thus, one acidic residue in the triglyceride holds two C_60_ fullerene molecules. It can be concluded that C_60_ forms crystal solvates with a very low solvent content with natural vegetable oils.

The structural peculiarities of investigated system were focused on the most probable distances between the fullerene molecule and the atoms of the triglyceride molecule. Table 1 shows the determined maxima of the radial distribution functions (RDF) between the fullerene molecule and triglyceride atoms. The fullerene molecule interacts to a greater extent with the hydrophobic hydrocarbon chains of carboxylic acid residues as compared with the atoms of the glycerol backbone and oxygen atoms of carbonyl groups. This is due to the hydrophobicity of hydrocarbon groups and fullerene.

**Table 1:** RDF maxima between fullerene and different triglyceride atoms.

*w* (C_60_)/%	C-Glycerine	O-Glycerine	O-Ol-Carbonyl	O-Pal-Carbonyl	Ol-Chain	Ol-non-saturated	Pal-Chain
4.0	6.91	6.73	6.85	6.11	6.09	5.21	4.55
5.3	6.13	6.39	6.57	6.85	5.71	4.31	4.45

C-Glycerine—carbon atoms of the glycerol backbone, O-Glycerine—oxygen atoms of the glycerol backbone, Ol-Chain—carbon atoms of the oleic acid residue, Pal-Chain—carbon atoms of palmitic acid residue, Ol-non-saturated—carbon atoms in the double bond of the oleic acid residue, O-Ol-Carbonyl—oxygen atoms in the carbonyl group of the oleic acid residue, O-Pal-Carbonyl—oxygen atoms in the carbonyl group of the palmitic acid residue.

**Figure 11:**
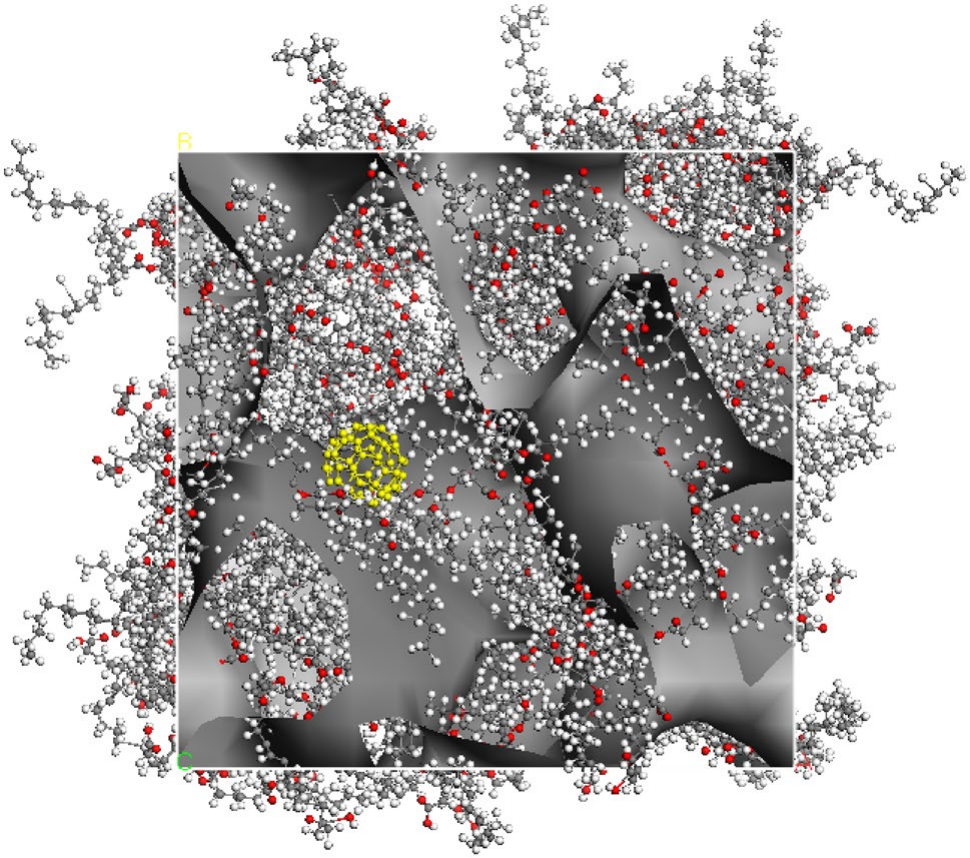
A density field of oleic residues in a system under study.

The residues of oleic and palmitic acids create a connected network for a sufficient movement of a fullerene molecule in olive oil. Density field of oleic residues is presented in Fig. 11.

The energy parameters of a simulated structure are presented in Table 2.

**Table 2:** The energy parameters of MD simulated structure.

Parameter	Initial	Final	Average	SD
Total energy/kcal·mol^−1^	1,077,990.513	18,785.248	19,196.299	4839.566
Potential energy/kcal·mol^−1^	1,063,459.137	4337.131	4664.912	3158.148
Kinetic energy/kcal·mol^−1^	14,531.377	14,448.117	14,531.387	1929.790
Total enthalpy	2,924,291.579	16,529.421	17,082.042	9749.331
Temperature/K	298.000	296.293	298.000	39.575
Pressure/GPa	76.700	− 0.094	− 0.088	0.220
Density/g·cm^−3^	0.920	0.920	0.920	0.000

As a result of the analysis of the mean-square displacements of fullerene molecules, it became possible to estimate the self-diffusion coefficients of fullerene in oil: 4.07·10^−7^ cm^2^·s^−1^ for a concentration of 4.0% and 2.85·10^−7^ cm^2^·s^−1^ for a concentration of 5.5%.

### Study of pharmacokinetics

**Figure 12:**
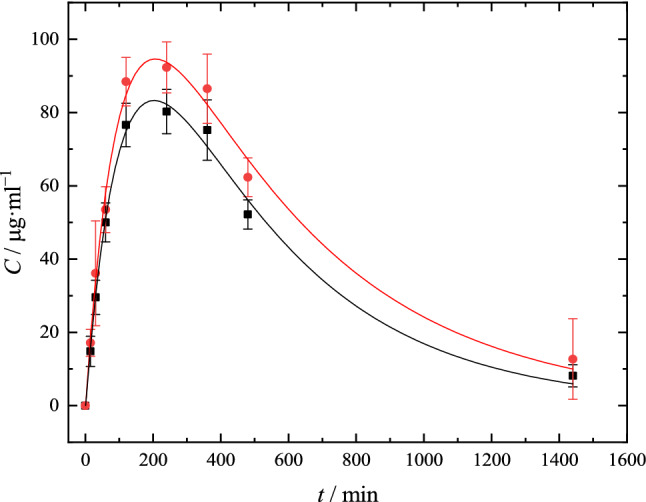
Pharmacokinetics of fullerene C_60_ in whole blood after oral administration to mice of an oil solution (–■–) and micellar form of fullerene C_60_ (–●–). Dots are experimental points; lines represent Eq. 2.

Administered C_60_ can be distributed between the blood, intercellular fluid and tissue cells. The distribution process largely depends on the relative affinity of C_60_ fullerene molecules and biomacromolecules of blood and tissues. Figure 12 shows the dependence of the content of fullerene C_60_ in whole blood on time, which was interpolated using ninth-order polynomial in PKSolver software^60^:

2 $$C_{p} (t) = \sum\limits_{i = 0}^{9} {a_{i} \cdot t^{i} } ,$$

where *a*_i_ (*i* = 0–9) are polynomial coefficients. The values of polynomial coefficients are summarised in Table 3.

**Table 3:** Calculated ninth-order polynomial coefficients for predicted curves of concertation time experimental values according to Eq. 3.

Substance	Polynomial coefficients									
	*a*_0_	*a*_1_	*a*_2_ × 10^3^	*a*_3_× 10^5^	*a*_4_× 10^8^	*a*_5_× 10^11^	*a*_6_× 10^14^	*a*_7_× 10^17^	*a*_8_× 10^21^	*a*_9_× 10^24^
Micellar C_60_	− 0.752	1.366	− 7.60	2.26	− 4.33	5.57	− 4.74	2.55	− 7.80	1.03
Oil C_60_	− 0.928	1.199	− 6.54	1.87	− 3.47	4.33	− 3.61	1.91	− 5.77	0.76

Based on experimental data (Fig. 12), PK parameters were calculated (Table 4). Area under the concentration time curve (AUC(0–t)) was calculated by ninth-order polynomial interpolation (see Eq. 2) and integration from zero time up to the last measured concentration:

3 $$AUC(0 - t) = \int\limits_{0}^{t} {C_{p} (t)dt} ,$$

where *C*_p_(*t*) is concentration time function.

**Table 4:** PK parameters of micellar and oil dispersions of C_60_ in conventional mice (*m* = 25 g) after oral administration of C_60_ in micellar and in oil forms (*n* = 10).

Parameter	Micellar C_60_	Oil C_60_
AUC (0–t)	1102.4 ± 189.1 mg h l^**−**1^	900.05 ± 104.2 mg h l^**−**1^
AUC (0–∞)	1184.7 ± 287.2 mg h l^**−**1^	941.14 ± 124.5 mg h l^**−**1^
MRT	10.00 ± 1.04 h	8.84 ± 1.12 h
*t*_1/2_	5.73 ± 0.71 h^**−**1^	4.83 ± 0.56 h^**−**1^
*t*_max_*	3.44 ± 0.31 h	3.38 ± 0.29 h
*C*_max_**	94.62 ± 6.95 mg l^**−**1^	83.23 ± 6.05 mg l^**−**1^
CL/F	1.2 10^**−**3^ ± 4 10^**−**4^ l kg h^**−**1^	0.016 ± 1.2 10^**−**3^ l kg h^**−**1^
Vz/F	0.010 ± 0.006 l kg^**−**1^	0.111 ± 0.010 l kg^**−**1^

*t_max_ is time of maximum plasma concentration.

**C_max_ is maximum plasma concentration.

Area under the concentration time curve extrapolated to infinite time (AUC (0–∞)) was calculated using Eq. 4:

4 $$AUC(0 - \infty ) = AUC(0 - t) + AUC(t - \infty ),$$

where AUC (t–∞) is area under the concentration–time curve from the last measured point to infinite time.

Mean residence time (MRT) represents the average time C_60_ molecule stays in the body; it was calculated using Eq. 5:

5 $$MRT = \frac{AUC(0 - t)}{{C_{p} (t = 0)}},$$

where *C*_p_ (*t* = 0) is the value of concentration after extrapolation to zero time.

Biological half-life (*t*_1/2_) is the time required for C_60_ to be reduced to half of its maximum concentration:

6 $$t_{1/2} = \frac{\ln 2}{{k_{e} }},$$

where *k*_e_ is elimination rate constant.

Oral clearance (CL/F) describes how efficiently C_60_ is eliminated when administered orally:

7 $$CL/F = \frac{D}{AUC(0 - \infty )},$$

where *D* is dose (mg kg^−1^).

Finally, apparent volume of distribution during terminal phase (V/F) was calculated using Eq. 8^61^:

8 $$V/F = \frac{CL/F}{{k_{e} }}.$$

Analysis of Table 4 reveals that, in both cases, C_60_ fullerene was found in the bloodstream achieving its highest concentration after 4 h after administration. In almost 5–6 h, C_60_ concentration decreases twice. It can be noted that biological half-life for C_60_ oil solution is less and it is eliminated faster than the micellar one. The comparison of all PK parameters reveals that the micellar form of C_60_ is more suitable as a basis for further development of prolongated nanocompositions.

Based on the results of Table 4, the bioavailability of the micellar form of fullerene C_60_ relative to the oil form was determined by the following equation:

9 $$F_{{{\text{rel}}}} = \frac{{AUC(0 - t)_{{{\text{micellar}}}} \cdot D_{{{\text{micellar}}}} }}{{AUC(0 - t)_{{{\text{oil}}}} \cdot D_{{{\text{oil}}}} }}.$$

Taking into account that the administered dose is 15 mg·kg^−1^ for C_60_ in oil and 1.5 mg·kg^−1^ for the micellar form, it follows that the micellar form is 12.2 times more bioavailable.

Figs. S1–S7 of the Supplementary Information and Table 5 demonstrate the dependence of the content of fullerene C_60_ on time in the lungs, thymus, heart, liver, spleen, kidneys and adrenal glands. It was found that fullerene C_60_, administered to mice in oil and micellar forms, appears in the blood of experimental mice for 15 min after its oral administration, reaching a maximum at 60 min and persists for 6 h. After 8 h, a decrease in the concentration of C_60_ and a further gradual a decrease up to a time of 24 h. Fullerene C_60_, administered to mice in oil and micellar forms, accumulates in the tissues of the liver, lungs, kidneys, heart, adrenal glands, thymus, and spleen. The detectable amounts of C_60_ fullerene are also determined 24 h after the administration of the oil solution and the micellar dispersion of C_60_ fullerene. The highest content of fullerene C_60_ was found in the liver and adrenal glands. It is important to note that fullerene C_60_, administered to mice in oil and micellar forms, was not detected in the brain tissue and testes. This is probably due to the presence of tissue barriers in these organs, namely blood–brain and blood–testis, respectively.

**Table 5:** Fullerene content in various organs after administration of an oil dispersion of fullerene to mice after 1 h (*C*_1_) and 24 h (*C*_24_) (data are given as mean ± standard deviation of the sample).

Organ	Form of administration of C_60_	*C*_1_/ng·g^−1^	*C*_24_/ng·g^−1^
Whole blood	Oil	50.00 ± 5.33	8.14 ± 3.02
	Micellar	53.50 ± 6.26	12.69 ± 10.99
Brain	Oil	Not found	Not found
	Micellar	Not found	Not found
Lungs	Oil	6.70 ± 0.90	11.30 ± 1.90
	Micellar	7.66 ± 1.03	7.99 ± 1.41
Thymus	Oil	9.84 ± 2.10	10.73 ± 2.50
	Micellar	10.65 ± 2.07	7.34 ± 2.07
Heart	Oil	14.39 ± 0.65	4.09 ± 1.64
	Micellar	14.22 ± 2.44	4.04 ± 0.50
Liver	Oil	36.82 ± 5.10	227.15 ± 14.73
	Micellar	69.01 ± 16.52	247.89 ± 38.20
Spleen	Oil	33.31 ± 4.72	74.81 ± 7.94
	Micellar	32.30 ± 6.74	86.04 ± 9.13
Kidneys	Oil	31.25 ± 1.52	17.37 ± 5.28
	Micellar	32.60 ± 7.31	19.98 ± 6.07
Adrenal glands	Oil	61.83 ± 5.01	51.35 ± 5.54
	Micellar	67.77 ± 10.62	26.72 ± 7.58
Testicles	Oil	Not found	Not found
	Micellar	Not found	Not found

In conclusion, we can say that since fullerenes have a number of unique properties, a relevant direction is the creation of systems for the delivery of these molecules and ways to increase their bioavailability. One of these forms can be the micellar form of C_60_ in olive oil, obtained using TWEEN-80. The prospect of studying C_60_ in olive oil was confirmed by the authors of ref.^62^, in which they carried out molecular and cytogenetic studies and showed that C_60_ in olive oil reduces CdCl_2_-induced genotoxicity in liver, kidney and bone marrow tissues of rats. Moreover, in ref.^63^ in experiments on erythrocyte integrity, platelet aggregation, and blood factors involved in coagulation, haemocompatibility of aqueous dispersions of C_60_ was revealed. On the other hand, analysis of the literature shows that fullerenes can be used as platforms for targeted drug delivery. For example, a conjugate based on C_60_ modified with glycine and docetaxel increases cellular uptake, efficacy of docetaxel and has an improved pharmacokinetic profile^64^.

## Conclusion

In this work, for the first time, studies on the biodistribution of fullerene C_60_ in two forms were carried out: a solution in olive oil and a micellar form under the ExtraOx trademark, created according to the original technology of “AQUANOVA RUS” JSC. As a result of the studies, it was found that the bioavailability of the micellar form of fullerene C_60_ increases 12 times compared to the oil form.

## Supplementary Information

Supplementary Information
