# Supplementary material for: Evaluation of the C60 biodistribution in mice in a micellar ExtraOx form and in an oil solution
Source: Sci Rep. 2021 Apr 16;11:8362. doi: 10.1038/s41598-021-87014-3 (PMC8052328; doi:10.1038/s41598-021-87014-3)
Supplement: Supplementary file 1 — Supplementary Information [file 41598_2021_87014_MOESM1_ESM.docx]

**Evaluation of the C_60_ biodistribution in mice in a micellar *ExtraOx* form and in an oil solution**

**Supplementary Information**

***Konstantin N. Semenov^a,b,с*^, Daria A. Ivanova^a^, Sergei V. Ageev^a,b^, Andrey V. Petrov^b^, Nikita E. Podolsky^b^, Ekaterina M. Volochaeva^d^, Ekaterina M. Fedorova^d^, Anatolii A. Meshcheriakov^a,b^, Egor E. Zakharov^a^, Igor V. Murin^b^, Vladimir V. Sharoyko^a,b,c*^***

*^a^*Pavlov First Saint Petersburg State Medical University, L’va Tolstogo ulitsa 6–8, Saint Petersburg, Russia, 197022

*^b^*Institute of Chemistry, Saint Petersburg State University, Universitetskii prospect 26, Saint Petersburg, Russia, 198504

*^с^*A. M. Granov Russian Research Centre for Radiology and Surgical Technologies, 70 Leningradskaya ulitsa, Saint Petersburg, 197758, Russia

*^d^*“AQUANOVA RUS” JSC, prospekt Nauki 12, Dubna, Moscow Oblast, Russia, 141983

^*^Corresponding author. E-mail address: knsemenov@gmail.com (K. N. Semenov), sharoyko@gmail.com (V.V. Sharoyko).

# Fig. S1. Pharmacokinetics of fullerene C_60_ in the lungs after oral administration to mice of an oil solution (–■–) and micellar form of fullerene C_60_ (–●–).

# Fig. S2. Pharmacokinetics of fullerene C_60_ in thymus after oral administration to mice of an oil solution (–■–) and micellar form of fullerene C_60_ (–●–).

# Fig. S3. Pharmacokinetics of fullerene C_60_ in the heart after oral administration to mice of an oil solution (–■–) and micellar form of fullerene C_60_ (–●–).

# Fig. S4. Pharmacokinetics of fullerene C_60_ in the liver after oral administration to mice of an oil solution (–■–) and micellar form of fullerene C_60_ (–●–).

# Fig. S5. Pharmacokinetics of fullerene C_60_ in the spleen after oral administration to mice of an oil solution (–■–) and micellar form of fullerene C_60_ (–●–).

# Fig. S6. Pharmacokinetics of fullerene C_60_ in the kidneys after oral administration to mice of an oil solution (–■–) and micellar form of fullerene C_60_ (–●–).В приложение

# Fig. S7. Pharmacokinetics of fullerene C_60_ in adrenal glands after oral administration to mice of an oil solution (–■–) and micellar form of fullerene C_60_ (–●–).
